# Does PAD and microcirculation status impact the tissue availability of intravenously administered antibiotics in patients with infected diabetic foot? Results of the DFIATIM substudy

**DOI:** 10.3389/fendo.2024.1326179

**Published:** 2024-05-07

**Authors:** Vladimíra Fejfarová, Radka Jarošíková, Simona Antalová, Jitka Husáková, Veronika Wosková, Pavol Beca, Jakub Mrázek, Petr Tůma, Jan Polák, Michal Dubský, Dominika Sojáková, Věra Lánská, Martin Petrlík

**Affiliations:** ^1^ Diabetes Centre, Institute for Clinical and Experimental Medicine, Prague, Czechia; ^2^ Department of Internal Medicine, Second Faculty of Medicine, Charles University, Prague, Czechia; ^3^ Department of Clinical Pharmacy and Drug Information Centre, Institute for Clinical and Experimental Medicine, Prague, Czechia; ^4^ Laboratory of Anaerobic Microbiology, Institute of Animal Physiology and Genetics, Czech Academy of Sciences, Prague, Czechia; ^5^ Department of Hygiene, Third Faculty of Medicine, Charles University, Prague, Czechia; ^6^ Department of Pathophysiology, Third Faculty of Medicine, Charles University, Prague, Czechia; ^7^ Vascular and Internal Medicine Outpatient Clinic, Prague, Czechia

**Keywords:** diabetic foot, antibiotic, infection, microdialysis, peripheral arterial disease

## Abstract

**Aims/hypothesis:**

The aim of this substudy (Eudra CT No:2019-001997-27)was to assess ATB availability in patients with infected diabetic foot ulcers(IDFUs)in the context of microcirculation and macrocirculation status.

**Methods:**

For this substudy, we enrolled 23 patients with IDFU. Patients were treated with boluses of amoxicillin/clavulanic acid(AMC)(12patients) or ceftazidime(CTZ)(11patients). After induction of a steady ATB state, microdialysis was performed near the IDFU. Tissue fluid samples from the foot and blood samples from peripheral blood were taken within 6 hours. ATB *potential* efficacy was *assessed by evaluating* the maximum serum and tissue ATB concentrations(C_max_ and C_max-tissue_)and the percentage of time the unbound drug tissue concentration exceeds the minimum inhibitory concentration (MIC)(≥100% _tissue_ and ≥50%/60% _tissue_ fT>MIC). Vascular status was assessed by triplex ultrasound, ankle–brachial and toe–brachial index tests, occlusive plethysmography comprising two arterial flow phases, and transcutaneous oxygen pressure(TcPO_2_).

**Results:**

Following bolus administration, the C_max_ of AMC was 91.8 ± 52.5 μgmL^-1^ and the C_max-tissue_ of AMC was 7.25 ± 4.5 μgmL^-1^(*P*<0.001). The C_max_ for CTZ was 186.8 ± 44.1 μgmL^-1^ and the C_max-tissue_ of CTZ was 18.6 ± 7.4 μgmL^-1^(*P*<0.0001). Additionally, 67% of patients treated with AMC and 55% of those treated with CTZ achieved tissue fT>MIC levels exceeding 50% and 60%, respectively. We observed positive correlations between both C_max-tissue_ and AUC_tissue_ and arterial flow. Specifically, the correlation coefficient for the first phase was *r=*0.42; (*P*=0.045), and for the second phase, it was *r*=0.55(*P*=0.01)and *r*=0.5(*P*=0.021).

**Conclusions:**

Bactericidal activity proved satisfactory in only half to two-thirds of patients with IDFUs, an outcome that appears to correlate primarily with arterial flow.

## Introduction

Diabetic foot (DF) is a serious late complication of diabetes that dramatically increases the risk of lower limb amputations (1). More importantly, it increases patient morbidity and mortality (2). Infection is one of the key components of DF, contributing to unfavorable patient prognosis and poor podiatric outcomes (3). Early diagnosis of diabetic foot infection (DFI), followed by prompt and aggressive therapy, has the potential not only to slow disease progression, but also to delay or even reverse the above complications (4).

For DFI management, podiatrists have several treatment modalities at their disposal. In mild forms of DFI, antiseptics or local devices with anti-infective substances can be effective in certain cases (5). For mild and moderate stages of DFI, antibiotics (ATBs) administered in various oral or intravenous regimens are recommended (6). For severe forms of DFI accompanied by sepsis, parenteral ATB therapy is strictly indicated (7).

ATBs are selected based on causative microbial agents and microbial sensitivity. To ensure an adequate antibacterial response, it is essential to administer ATBs at levels conducive to optimal bactericidal activity in both serum and peripheral tissues (8). However, the efficacy of ATBs, particularly in peripheral tissues, can be affected by several factors. We hypothesize that macro- and microangiopathy are the most influential of these. However, researchers have yet to comprehensively address the serum and tissue concentrations of time-dependent ATBs, that are not routinely monitored in patients with DFI, especially those suffering from peripheral arterial disease (PAD). Therefore, the aim of our study was to assess the availability and bactericidal effect of ATBs in patients with infected diabetic foot ulcers (IDFUs) in the context of micro- and macrocirculation status.

## Research design and methods

### Study participants

A total of 23 patients with type 2 diabetes mellitus (DM) and IDFUs, graded (2–3)–(0–3)–(2–3) according to the WIfI classification (9), were enrolled in a substudy of the DFIATIM *(Diabetic Foot Infection treated with ATBs and it’s Impact on gut Microbiota)* single-center randomized prospective comparative trial (**Table 1**). DFU infection was determined based on the following parameters: clinical signs, including phlegmon, edema, redness, pathological ulcer secretion, deepening of the DFU, and fetor; laboratory markers of infection, such as CRP and leukocytosis; positive bacterial findings in tissue samples or swabs taken from the base of the IDFU after debridement; and in several cases positive probe-to-bone test (10) and/or positive bone biopsy results. DFI was categorized as mild, moderate, or severe based on the Infectious Diseases Society of America (IDSA) criteria (7). Patients aged 30 to 75 years suffering from moderate or severe DFI caused by Gram-positive or Gram-negative bacteria sensitive to either amoxicillin/clavulanic acid (AMC) or ceftazidime (CTZ) were enrolled in this substudy of the DFIATIM trial. Exclusion criteria included severe hepatic insufficiency, chronic renal insufficiency/failure corresponding to stages 4 and 5 of the Chronic kidney disease (CKD) classification, severe malnutrition, indication for emergent foot amputation, recent percutaneous transluminal angioplasty (within 2 weeks), indication for acute revascularization due to rapid progression of PAD or acute arterial ischemia, allergy to test ATBs, presence of a diagnosed neoplasm, pregnancy, lactation, septic shock, active Epstein–Barr virus, inflammatory bowel disease, celiac disease or any other malabsorption disease, and acute gastroenteritis.

**Table 1:** Basal characteristics of study participants and their circulation status.

Evaluated parameters	Patients treated with bolus AMC therapy	Patients treated with bolus CTZ therapy	*P* value
Number of patients	12	11	
Age (years)	60.1 ± 6.9	65.3 ± 8.5	NS
Weight (kg)	99.1 ± 16.8	103.5 ± 18.4	NS
BMI (kg m^-2^)	30.1 ± 4.9	31 ± 4	NS
HbA1c (mmol mol^-1^)	59.7 ± 15.4	63.4 ± 16.2	NS
CRP (mg L^-1^)	17.3 ± 25.1	49.8 ± 65	NS
Serum albumin (g L^-1^)	40.23 ± 3.6	40.15 ± 5.3	NS
Serum creatinine (μmol L^-1^)	98.2 ± 40.8	90 ± 24.9	NS
Glomerular filtration (CKD-EPI) (mL s^-1^ × 1.73 m^-2^)	1.27 ± 0.5	1.29 ± 0.5	NS
ABI	1.03 ± 0.32	1.01 ± 0.38	NS
TBI	0.66 ± 0.19	0.7 ± 0.27	NS
PAD (% of study participants)	44%	70%	NS
Arterial flows in first phase (mL *min^-^* ^1^)	78.3 ± 42.9	79.4 ± 27.4	NS
Arterial flows in second phase (mL *min^-1^*)	37.9 ± 18.2	48.5 ± 20.4	NS
Interarm distance (mm)	23.9 ± 4.1	25.6 ± 3.5	NS
TcPO_2_ (mm Hg)	43 ± 12.6	47 ± 12.4	NS

All results are presented as means ± standard deviation; BMI, body mass index; HbA1c, glycated hemoglobin; CRP, C-reactive protein; AMC, amoxicillin/clavulanic acid; CTZ, ceftazidime; CKD, chronic kidney disease; NS, nonsignificant; ABI, ankle-brachial index; TBI, toe-brachial index; PAD, peripheral arterial disease; TcPO_2_, transcutaneous oxygen pressure.

Prior to enrolment in the study, all patients signed informed consent forms, which were approved by the ethics committees of the Institute for Clinical and Experimental Medicine and Thomayer University Hospital (both Prague, Czech Republic).

### Vascular status assessment

#### Large vessel evaluation

During the inclusion visit, all study participants were evaluated for both macrocirculation (larger vessels) and microcirculation status. Assessment of peripheral arterial circulation consisted of foot pulse measurement and triplex ultrasound of the peripheral arteries (11, 12). Additionally, we measured systolic blood pressure in the peripheral arteries, including the dorsalis pedis artery, the posterior tibial artery, using a handheld Doppler ultrasound device (Edan SD3 Vascular Ultrasonic Pocket Doppler, EdanUSA, San Diego, CA) equipped with an 8 MHz probe. The same technique was used to evaluate the ankle–brachial index (ABI) and toe–brachial index (TBI) (12). See **Table 1** for details.

Color-coded triplex ultrasound has emerged as the gold standard in the field of accurate PAD detection. For this purpose, we used the LOGIQ P7 ultrasound system (GE Healthcare) equipped with a 4 MHz or 8 MHz probe, operating at the factory default setting. To determine morphology and blood flow in the peripheral limb arteries, pulse wave correction was set to the standard angle of 70 degrees followed by appropriate adjustments to the pulse repetition frequency. Monophasic waves were used to identify the presence of hemodynamically significant stenosis or obliteration. Arterial lesions that modify pulse waveforms are considered to have clinical hemodynamic significance due to their ability to dramatically reduce peripheral perfusion.

#### Arterial flow volume

The volume of arterial flow in the treated lower limb was measured using occlusive plethysmography (OP), a noninvasive diagnostic tool for screening PAD and evaluating vessel functionality. Using air plethysmography and photoplethysmography, we are usually able to detect the quality of perfusion in the peripheral lower limb. During the arterial phase of OP, conducted using the VLab-4000 plethysmography device (Advanced Medical Solutions, Czech Republic), the volume of arterial flow was measured in two phases - first and second phase. We also evaluated another parameter, the interarm distance. If the interarm distance is found to be elongated during arterial flow measurement, this can indicate similarly as reduced first or second phase of arterial flow stenosis or obliteration of the proximal arteries (**Table 1**). The probability of PAD significantly increased with lengthening of the interarm distance. The interarm distance ≥ 20 mm is more reproducible, values greater than 25 mm attain a sufficiently high positive predictive value for PAD diagnosis. The detection of interarm distance of pulse wave together with the change of pulse wave shape are crucial for interpretation of the presence or absence of PAD detected by occlusive plethysmography. This interarm distance is counted based on Oliva-Roztočil index (it is defined as the distance between ascending and descending components of the arterial pulse wave, measured at two-thirds of the amplitude of the pulse; (13), the relatively old method described in 1983 (14). Based on angiology experts, this interarm distance is still used during the evaluation of different plethysmographic methods results (15).

The usage of occlusive plethysmography is relatively widely enlarged especially in angiology centres in middle Europe. This method belongs to time- (duration of plethysmography assessment is 15 minutes) and financially saving assessment (1/3 evaluation ultrasound cost) in contrast to ultrasound methods. There are no limitations to be performed in infected or ischemic feet.

#### Transcutaneous oxygen measurement

Microcirculation status was determined by measuring transcutaneous oxygen pressure (TcPO_2;_ **Table 1**). TcPO_2_ was measured using the TINA TCM 400 transcutaneous monitoring system (Radiometer, Copenhagen) equipped with Clark electrode. We used this device to electrochemically determine the partial pressure of oxygen on the surface of the skin (12, 16). This method consisted of heating a standard probe featuring a small chamber containing silver and platinum electrodes with an oxygen-permeable membrane. The temperature was raised to 42 - 45°C for arterialized cutaneous flow, thus increasing oxygen diffusion through the skin via local vasodilation. The results obtained were automatically recalculated to 37°C (16).

### DFIATIM clinical trial

**Figure 1:**
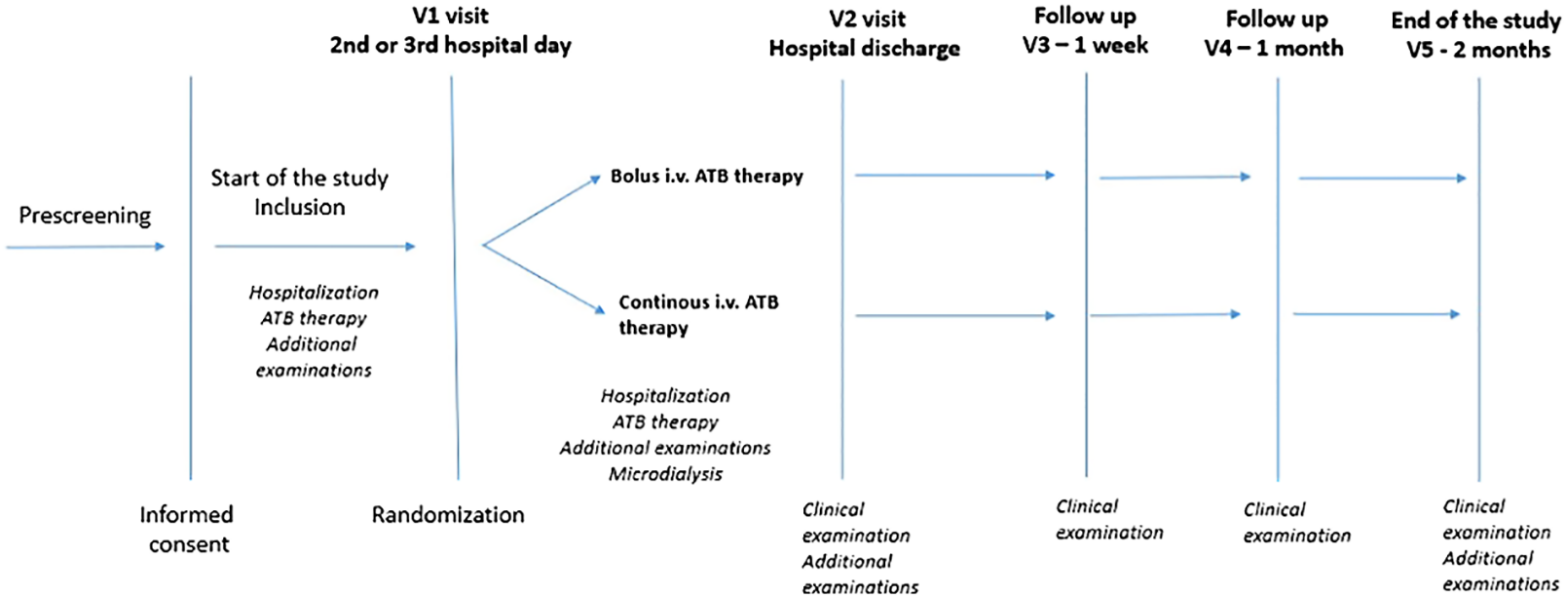
DFIATIM study scheme.

This article provides data pertaining to a substudy of the DFIATIM clinical trial. In this single-center randomized prospective comparative trial, 60 patients with infected DF meeting the inclusion and exclusion criteria will be enrolled for treatment with intravenous ATBs (sample size estimation see statistics). The cohort will be divided into two groups. The first group, comprising 30 patients with infections caused by pathogens sensitive to the CTZ, will receive intravenous CTZ treatment. The second group, consisting of 30 individuals infected with bacteria sensitive to AMC, will receive intravenous AMC treatment. After the initial inclusion visit, patients will be admitted to hospital. Parenteral administration of AMC (1.2 g every 8 hours) or CTZ (2.0 g every 8 hours) will be initiated using standard bolus regimens. This will induce a steady state of ATBs consisting of at least 5 applications. Randomization, performed in each study arm in a 1:1 ratio according to the prescribed scheme, will determine whether patients receive ATBs in bolus or continuous dosage patterns. Serum and tissue concentrations of ATBs will then be monitored via blood and microdialysis (MD) sampling. Until discharged from hospital, patients will be treated according to different ATB dosage regimens in line with the randomization scheme (**Figure 1**). The total duration of intravenous ATB therapy will last 6 - 7 days. After hospital discharge, patients will be followed and undergo standard treatment according to the study schedule (**Figure 1**). The DFIATIM trial has been approved by the ethics committees of the Institute for Clinical and Experimental Medicine and Thomayer University Hospital.

Only patients treated with intravenously administered ATBs using the bolus method were included in the substudy presented in this article. Of these, 12 patients received AMC treatment and 11 were treated with CTZ.

### Microdialysis

After achieving a steady state of ATB administration involving 5 bolus applications while in hospital, patients underwent microdialysis. During this procedure, tissue fluid and blood samples were collected simultaneously over a period lasting a minimum of six hours. The aim of the procedure was to measure the concentrations of ATBs in tissue fluid.

Microdialysis is a minimally invasive sampling technique designed primarily for *in vivo* monitoring of metabolic, biochemical, physiological, and pharmacological processes in living tissues and organs (17). It is widely used in pharmaceutical research, particularly for assessing drug pharmacokinetics and pharmacodynamics in peripheral tissues (17). Microdialysis enables *in vivo* monitoring of small water-soluble substances in the extracellular environment of tissues. Therefore, microdialysis serves as an ideal diagnostic tool for evaluating levels of tissue ATBs (18).

In this study, we employed the 63 Microdialysis Catheter (CMA Microdialysis AB, Stockholm, Sweden), which features a semipermeable hollow membrane. The catheter was connected to a syringe pump via an inlet tube for saline solution and an outlet tube for collecting microdialysate. In our patients with IDFUs, the probe was inserted into the subcutaneous tissue near the wound under antiseptic conditions and then flushed with perfusate solution at a constant flow rate of 0.5 to 5.0 μL min^-1^ (17). The microdialysis catheter was connected to a microperfusion pump and perfused with Ringer’s solution. Briefly, the perfusate rinses the membrane from the inside and, as it flows through the probe, becomes enriched with ATBs, which are then transported from the tissue across the membrane into the perfusate based on their concentration gradient (19). The outflowing microdialysate was collected in an Eppendorf tube at intervals of 30 min. The catheter was then removed under aseptic conditions and discarded. All microdialysate samples were frozen immediately and stored at -80°C until analysis.

To optimize microdialysis recovery, expressed as the ratio between the concentration of the substance in the microdialysate and its concentration in the tissue, we used a microdialysis probe with a cut-off at least 4 times higher than the molecular weight of the ATBs (18). Ringer’s solution was used as a perfusion medium to simulate the composition of extracellular fluid and minimize undesired transport of substances into and out of the membrane due to differences in osmolarity (20).

Before commencing this DFIATIM substudy, we performed retrodialysis to establish the technical recovery, or transition ability of ATBs across the microdialysis membrane (19). Measurements were carried out under laboratory conditions using samples of human serum obtained from healthy volunteers (21). We have chosen such perfusion rate based on previous in vitro and in vivo repeated tests with low interindividual variability to find optimal perfusion rate to recovery sufficient volume of tissue samples and achieve ATB molecules transmission into the microdialysis canula and collector. To achieve a high microdialysis recovery, expressed as the ratio between the concentration of the substance in the microdialysate versus the concentration in the tissue, it is necessary to use a cut-off that is 4 times higher compared to molecular weight (22).

In our study, approximately 0.5 mL of serum was spiked with AMC or CTZ at two concentration levels: 10 and 40 μg mL^-1^. Next, the microdialysis probe was inserted into the spiked solution in a plastic vessel and rinsed with Ringer’s solution at a flow rate of 2.5 μL min^-1^. After attaining a steady-state flow rate, microdialysis samples were collected over a period of 30 min. The spiked serum samples obtained were then analyzed by capillary electrophoresis (CE). The relative concentration levels were used to calculate the microdialysis recovery, which were 76.2% for AMC and 81.1% for CTZ. Importantly, these values align with previously reported microdialysis yields of ATBs determined by retrodialysis using a probe inserted into the subcutaneous tissue (20). The values were used to recalculate the ATB levels detected in the microdialysis samples obtained from patients, adjusting them to reflect concentrations in subcutaneous tissue.

### Capillary electrophoresis

The relatively small volumes of clinical samples available for analysis require the use of a suitable microanalytical technique (23–26) to allow sensitive quantitative monitoring of ATBs over short time intervals. The most common are highly selective biosensors or mass spectrometry methods combined with direct sample injection or high-performance liquid chromatography (HPLC). Since both serum and microdialysates of tissues are highly complex biological matrices, it is advisable to employ an efficient separation technique such as capillary electrophoresis (CE) (27–30). Due to the minimal requirements on the amount of clinical material and its laboratory processing, CE represents a suitable tool for the analysis of microliter quantities of collected microdialysates. These small volumes can be injected directly into the electrophoretic capillary after only a one-step dilution with organic solvent, providing an efficient sample pretreatment method.

The separation in CE is controlled by an electric field of high intensity up to 1 kV cm^-1^, which results in relatively short ATB migration times lasting only a few minutes. In addition, in combination with universal contactless conductivity detection (C^4^D) (31), the need for sample derivatisation is eliminated, and ATBs are determined directly in their native forms in which they are found in the human body.

For this study, CE-C^4^D determination of AMC and CTZ was performed in off-line mode. This involved collecting serum and microdialysis samples at the clinical workplace, freezing them, and transporting them to the analytical laboratory. The thawed clinical samples, ranging in volume between 15 - 20 μL, were mixed with three times the volume of acetonitrile as an organic solvent that serves to deproteinise the clinical sample and simultaneously suppresses its electrical conductivity. After centrifugation, the supernatant is immediately analyzed by CE-C^4^D; details are summarized in our recent publications (19, 21, 29).

### Efficacy of ATB therapy

The efficacy of ATB therapy in patients with IDFUs depends on achieving a certain level of bactericidal activity. This is evaluated using the following parameters: maximum serum concentration (C_max_) and maximum tissue concentration (C_max-tissue_); the ratio of C_max_ or C_max-tissue_ to the minimum inhibitory concentration (MIC) of the causative agents (C_max_/MIC and C _max-tissue_/MIC); the ratio of the area under the serum or tissue concentration–time curve (AUC or AUC_tissue_) to the MIC (AUC/MIC and AUC_tissue_/MIC); and the duration of the dosing interval during which plasma or tissue concentration exceeds the MIC (for AMC, ≥ 50% and ≥ 100% fT > MIC and ≥ 50% and ≥ 100% _tissue_ fT > MIC; for CTZ, ≥ 60% and ≥ 100% fT > MIC and ≥ 60% and ≥ 100% _tissue_ fT > MIC; 32). The measurements of 24 hours AUC were conducted using the trapezoid method over an extended eight-hour interval. It was counted in the following manner: based on pre-administration concentration C0 (C through), which was collected in a steady state, we determine 24 hours AUC using the rule of three since we assume that before the next administration, the concentration will remain stable.

The efficacy of ATB therapy depends on achieving effective ATB concentrations, when the levels of monitored ATBs at least reach, better exceed the MIC of the causative pathogens for at least half (in the case of AMC)/60% of the daily time (in the case of CTZ), preferable for 100% of the time when we administer ATB to a patient with infected diabetic foot. This is the most effective way how to prevent infection in diabetic foot.

The aim of this study was to establish the impact of various factors, including the presence of PAD defined by triplex ultrasound or facultatively by angiography, computed tomography angiography, magnetic resonance angiography, ABI, TBI, and occlusive plethysmography and microcirculation status, determined by TcPO_2_ for key parameters such as bactericidal activity.

### Statistical analysis

Data are expressed as the mean ± standard deviation or the median and range for continuous variables, and as relative frequencies for discrete variables. Data were tested for normality using the Shapiro-Wilk test. Parametric tests were used for normally distributed data, while nonparametric methods were used for data that were not normally distributed. Differences between the two groups were compared using the *t-*test for normal distribution or the Mann-Whitney test for other types of continuous distribution. Discrete variables were tested by χ^2^-test. Pearson (normal) and Spearman (other) correlation coefficients were used to measure the associations between variables. Dependent samples were tested by the one-sample Wilcoxon signed rank test. All tests were two-sided and a *P* value < 0.05 was considered statistically significant. *Significance levels after Holm’s correction for multiple testing (PH) are also presented.* Statistical analysis was performed using JMP^®^16.2.0 statistical software (SAS Institute, Cary, NC). Statistical processing was conducted using standard parametric and nonparametric methods in collaboration with statisticians at the Institute for Clinical and Experimental Medicine, Prague.

## Results

The study cohorts did not differ significantly in basal characteristics, including circulation parameters (**Table 1**). DFUs were infected by 2 to 3 pathogens on average (2.5 ± 0.9 microorganisms per DFU in the AMC group vs. 2.5 ± 1.4 bacteria per DFU in the CTZ group; NS). However, there were variations in the MIC of the causative agents (0.54 ± 0.54 vs. 4.68 ± 3.2; *P* = 0.002; *(PH=0.008);* **Table 2**).

**Table 2:** *Pharmacokinetic parameters* in relation to bactericidal activity.

Evaluated parameters	Patients treated with bolus AMC therapy	Patients treated with bolus CTZ therapy
Number of patients	12	11
C_max_ (μg mL^-1^)	91.8 ± 52.5	186.8 ± 44.1
C_max-tissue_ (μg mL^-1^)	7.25 ± 4.5 *	18.6 ± 7.4**
24 hours AUC	238.7 ± 78.7	1077 ± 344
24 hours AUC_tissue_	65.5 ± 34.8	226.2 ± 79.1
MIC of causative microbial agent	0.54 ± 0.54	4.68 ± 3.2***
AUC/MIC	1170 ± 1304	556 ± 580
AUC_tissue_/MIC	334 ± 351	113.3 ± 114.3

All results are presented as means ± standard deviation; *Comparison between C_max_ and C_max-tissue_ AMC values (P = 0.0001–0.036; PH=0.0004 - >0.1). **Comparison between C_max_ and C_max-tissue_ CTZ values (P = 0.0001; PH=0.0004). ***Comparison between MIC values of causative agents detected in the group of patients treated with AMC and those treated with CTZ (P = 0.002; PH=0.008). C_max_, maximum concentration; AUC, area under the curve; MIC, minimum inhibitory concentration.

**Figure 2:**
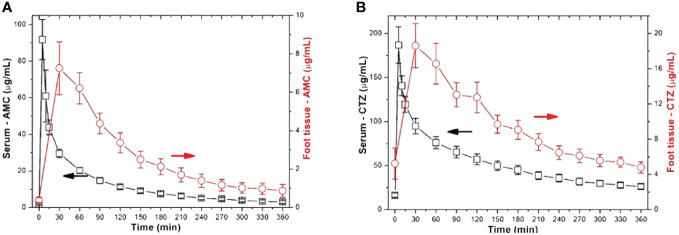
Comparison of serum and tissue ATB concentrations in study participants with IDFUs (**A** – AMC; **B** -CTZ).

Concerning the assessment of pharmacokinetic parameters for AMC, the maximum serum levels of AMC (C_max_) after bolus administration peaked at around 91.8 ± 52.5 μg mL^-1^. AMC concentrations in peripheral tissues (C_max-tissue_) reached approximately 9 – 31% of serum levels (ranging from 0.78 ± 0.97 to 7.3 ± 4.5 μg mL^-1^; *P* = 0.0001 - 0.036; *(PH=0.0004 - >0.1;* **Figure 2A**).

**Figure 3:**
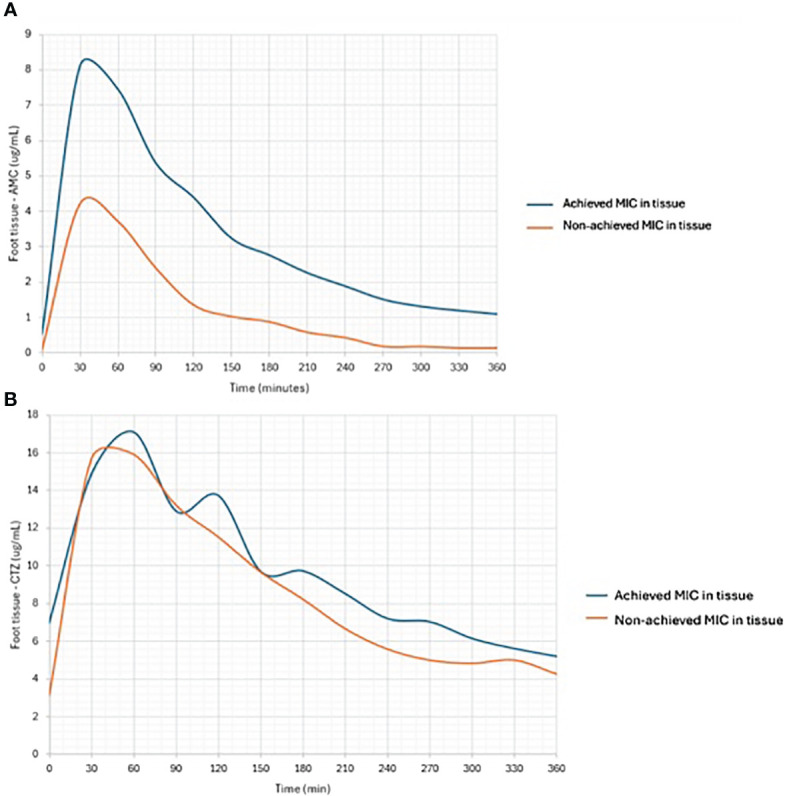
Comparison of tissue concentrations of AMC **(A)** and CTZ **(B)** in subjects achieving or non-achieving MIC in foot tissue samples.

When examining the area under the concentration–time curve for plasma or tissue, these microbicidal parameters proved relatively satisfactory. Similarly, when comparing the same parameters with MIC values, their ratios were within an acceptable range (**Table 2**). *To treat DFI effectively*, more than half of the daily tissue ATB concentrations must be greater than the MIC of the causative microbial agent. Among AMC-treated patients, only 67% had tissue concentrations that were above the MIC for at least 50% of the time (≥ 50% _tissue_ fT > MIC*;* **Figure 3A** *)*. In contrast, only 33% of patients with IDFUs maintained tissue concentrations above the MIC for at least 100% of the time (≥ 100% _tissue_ fT > MIC).

Patients treated with CTZ reached maximum serum CTZ levels of almost 187 μg mL^-1^. In patients with IDFUs treated with CTZ, the maximum peripheral tissue concentration was 18.6 ± 7.4 μg mL^-1^. Compared to serum levels, tissue CTZ levels ranged between 4.8 ± 2.1 and 18.6 ± 7.4 μg mL^-1^; *P* = 0.0001; *(PH=0.0004);* **Figure 2B**), reaching approximately 9 – 22% of serum CTZ concentrations. Other pharmacological parameters detected in patients from the CTZ group are given in **Table 2**. Similar to the study participants treated with AMC, only 55% patients achieved more than half of the daily tissue CTZ concentrations higher than the MIC of pathogens (**Figure 3B**). Of the CTZ-treated patients, 45% had ≥ 100% _tissue_ fT > MIC.

**Figure 4:**
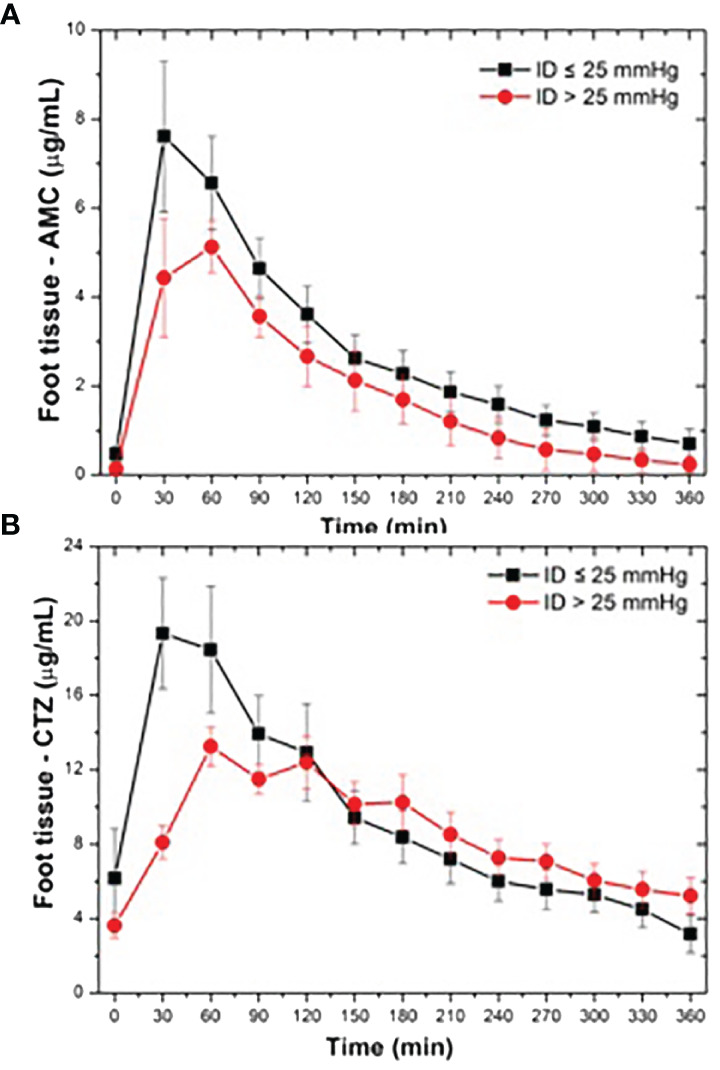
Comparison of tissue concentrations of AMC **(A)** and CTZ **(B)** in study subjects based on achieved Interarm distance detected during occlusive plethysmography (with cut-point 25 mm Hg).

We identified positive and negative correlations of ATB bactericidal activity markers with some patient characteristics, particularly renal functions and body weight (**Table 3**). Regarding circulation parameters, only arterial flow correlated significantly with tissue ATB concentrations. The correlation coefficient for the first arterial phase was *r* = 0.42 (*P* = 0.045; *(PH - >0.1)*), and for the second phase, it was *r* = 0.44 – 0.55 (*P* = 0.0463 - 0.01*; PH>0.1 - >0.1*). Other macro- and microcirculation determinants *(including plethysmographic interarm difference with cut-point of 25 mm*, **Figures 4A, B** *)* did not correlate significantly with *pharmacokinetic parameters*.

**Table 3:** Correlations between markers of ATB bactericidal activity and patient characteristics, including vascular parameters.

Markers of ATB bactericidal activity	Patient characteristics	Spearman r (95% CI), Pearson r value	p- value
*C_max_*	*Age*	*0.135*	*NS*
*C_max_*	*BMI*	*0.046*	*NS*
*C_max_*	*Weight*	*-0.031*	*NS*
*C_max_*	*HbA1c*	*-0.137*	*NS*
*C_max_*	*ABI*	*0.062*	*NS*
*C_max_*	*TBI*	*0.211*	*NS*
*C_max_*	*TcPO2*	*0.403*	*NS*
*C_max_*	*First phase of arterial flow*	*-0.009*	*NS*
*C_max_*	*Second phase of arterial flow*	*0.088*	*NS*
*C_max_*	*Interarm distance*	*0.251*	*NS*
*C_max_*	*Serum creatinine*	*0.141*	*NS*
*C_max_*	*GF*	*-0.171*	*NS*
*C_max-tissue_*	*Age*	*0.062*	*NS*
*C_max-tissue_*	*BMI†*	*-0.557*	*p=0.06*
*C_max-tissue_*	*Weight*	*-0.195*	*NS*
*C_max-tissue_*	*HbA1c*	*0.012*	*NS*
*C_max-tissue_*	*ABI*	*0.217*	*NS*
*C_max-tissue_*	*TBI*	*-0.056*	*NS*
*C_max-tissue_*	*TcPO2*	*-0.056*	*NS*
*C_max-tissue_*	*First phase of arterial flow§*	*0.421(0.0111 to 0.71)*	*p=0.045*
*C_max-tissue_*	*Second phase of arterial flow§*	*0.548(0.152 to 0.792)*	*p=0.01*
*C_max-tissue_*	*Interarm distance*	*0.199*	*NS*
*C_max-tissue_*	*Serum creatinine*	*0.136*	*NS*
*C_max-tissue_*	*GF*	*-0.115*	*NS*
*24 hours AUC*	*Age*	*0.33*	*NS*
*24 hours AUC*	*BMI†*	*-0.55*	*p=0.06*
*24 hours AUC*	*Weight†*	*-0.557*	*p=0.06*
*24 hours AUC*	*HbA1c*	*0.09*	*NS*
*24 hours AUC*	*ABI*	*0.13*	*NS*
*24 hours AUC*	*TBI*	*-0.023*	*NS*
*24 hours AUC*	*TcPO2*	*0.105*	*NS*
*24 hours AUC*	*First phase of arterial flow*	*0.171*	*NS*
*24 hours AUC*	*Second phase of arterial flow§*	*0.439 (0.0095 to 0.732)*	*p=0.046*
*24 hours AUC*	*Interarm distance§*	*0.407*	*p=0.06*
*24 hours AUC*	*Serum creatinine†*	*0.923 (0.743 to 0.979)*	*p<0.001*
*24 hours AUC*	*GF†‡*	*-0.825 (-0.949 to -0.477)*	*p=0.001*
*24 hours AUC_tissue_*	*Age*	*0.307*	*NS*
*24 hours AUC_tissue_*	*BMI†*	*-0.585(-0.868 to -0.0166)*	*p=0.046*
*24 hours AUC_tissue_*	*Weight*	*-0.079*	*NS*
*24 hours AUC_tissue_*	*HbA1c*	*0.063*	*NS*
*24 hours AUC_tissue_*	*ABI*	*0.151*	*NS*
*24 hours AUC_tissue_*	*TBI*	*-0.063*	*NS*
*24 hours AUC_tissue_*	*TcPO2*	*0.062*	*NS*
*24 hours AUC_tissue_*	*First phase of arterial flow*	*0.286*	*NS*
*24 hours AUC_tissue_*	*Second phase of arterial flow§*	*0.501(0.0886 to 0.767)*	*p=0.046*
*24 hours AUC_tissue_*	*Interarm distance*	*0.346*	*NS*
*24 hours AUC_tissue_*	*Serum creatinine*	*0.204*	*NS*
*24 hours AUC_tissue_*	*GF*	*-0.217*	*NS*
*AUC/MIC*	*Age*	*-0.007*	*NS*
*AUC/MIC*	*BMI*	*-0.27*	*NS*
*AUC/MIC*	*Weight*	*-0.407*	*NS*
*AUC/MIC*	*HbA1c*	*0.003*	*NS*
*AUC/MIC*	*ABI*	*0.319*	*NS*
*AUC/MIC*	*TBI*	*-0.418*	*NS*
*AUC/MIC*	*TcPO2*	*-0.024*	*NS*
*AUC/MIC*	*First phase of arterial flow*	*0.038*	*NS*
*AUC/MIC*	*Second phase of arterial flow*	*0.0492*	*NS*
*AUC/MIC*	*Interarm distance*	*0.249*	*NS*
*AUC/MIC*	*Serum creatinine*	*0.107*	*NS*
*AUC/MIC*	*GF*	*-0.039*	*NS*
*AUC_tissue_/MIC*	*Age*	*0.035*	*NS*
*AUC_tissue_/MIC*	*BMI†*	*-0.815 (-0.955 to -0.38)*	*p=0.004*
*AUC_tissue_/MIC*	*Weight†§*	*-0.6687 (-0.914 to -0.068)*	*p=0.035*
*AUC_tissue_/MIC*	*HbA1c*	*-0.034*	*NS*
*AUC_tissue_/MIC*	*ABI*	*0.35*	*NS*
*AUC_tissue_/MIC*	*TBI*	*-0.359*	*NS*
*AUC_tissue_/MIC*	*TcPO2*	*-0.102*	*NS*
*AUC_tissue_/MIC*	*First phase of arterial flow*	*0.057*	*NS*
*AUC_tissue_/MIC*	*Second phase of arterial flow*	*0.041*	*NS*
*AUC_tissue_/MIC*	*Interarm distance*	*0.187*	*NS*
*AUC_tissue_/MIC*	*Serum creatinine*	*0.112*	*NS*
*AUC_tissue_/MIC*	*GF*	*-0.034*	*NS*

†Significant correlations only in the AMC group. ‡ Significant correlations only in the CTZ group. § Significant correlations in all patients treated with AMC or CTZ. C_max_, maximum concentration; AUC, area under the curve; BMI, body mass index; MIC, minimum inhibitory concentration; GF, glomerular filtration.

## Conclusions

The maximum effect of ATB therapy in iDFUs depends on achieving serum and tissue concentrations that provide effective bactericidal action. The efficacy of ATB therapy is typically assessed based on clinical evaluation during podiatric care, rarely using diagnostic methods. Serum ATB levels are usually only monitored for dose-dependent ATBs such as amikacin and gentamicin. However, time-dependent ATBs used to treat DFIs, including beta-lactams and cephalosporins (32), are not routinely assessed in the laboratory. In fact, there are almost no data on the pharmacokinetic and pharmacodynamic effects of these ATBs on the serum and peripheral tissues of DF patients (33).

The DFIATIM study aims not only to fill this knowledge gap, but also to test a number of clinical hypotheses. One of these is that PAD or microcirculatory impairment significantly affects the bactericidal activity and tissue availability of administered ATBs, especially in the DF. The potential influence of PAD on the accessibility of time-dependent ATBs in the lower limbs has yet to be addressed in the literature. Microdialysis is the most suitable method for clearly determining tissue concentrations of ATBs, monitoring their pharmacokinetic and pharmacodynamic properties, and ensuring the efficacy of intravenous ATB administration in patients with infected DF and PAD (29, 34). Before the introduction of microdialysis into clinical research, ATBs were detectable only in samples obtained from surgical resections or amputations, including even bone material (34, 35). Microdialysis facilitates real-time *in vivo* monitoring of ATBs (34). This is largely due to the use of a porous catheter membrane, which allows ATBs to diffuse across the membrane into the catheter according to the concentration gradient and the molecular weight of ATBs. We have previously validated this technique using our own *in vitro* and *in vivo* experiments (19).

Several methods, each with its own advantages and disadvantages, can be used to determine the concentrations of ATBs in serum and tissue. For our ongoing DFIATIM trial, we use a modern analytical method based on CE–C^4^D (23) to sensitively determine ATBs in microdialysis samples (35). Compared to conventional HPLC techniques, CE approaches to ATB monitoring in microdialysis offer greater insights for several reasons (36). The amount of the sample injected into the separation capillary varies in nanoliter volumes, forming a complementary technique intended for tissue sampling by microdialysis. CE–C^4^D enables direct measurement of a wide range of ATBs in their native states, bypassing the need for complex derivatization reactions (37) or sample treatments that are difficult to perform in microliter amounts. The short migration times of electrophoretic analysis (38) are essential for continuous monitoring of microdialysates, particularly in pharmacological and physiological studies (39). Our previous study clearly demonstrated that the electrophoretic stacking technique is suitable for accurate determination of cephalosporin CTZ and beta-lactam AMC in the blood plasma and microdialysate of foot tissue after ATB administration (19). The concentrations we obtained were in close agreement with a conventional HPLC technique (40).

After incorporating new microdialysis and CE methods within our podiatric practice, we decided to carry out the prospective DFIATIM trial to elucidate the time-dependent concentrations of ATBs in serum and tissue as well as their bactericidal activities. We were particularly interested to discover in one substudy of mentioned trial how the vascular status of the study participants could influence these parameters.

Our results revealed that bolus intravenous administration of both AMC and CTZ yielded satisfactory serum ATB levels. Similarly, Gariani et al. confirmed the satisfactory clinical impact of oral AMC on DFI, similar to clinical outcomes for other antimicrobial regimens (41). However, data on CTZ are practically non-existent in the literature. The bactericidal activity of ATBs are determined by the time interval over which ATB concentrations in tissue exceed the MIC. For AMC, tissue concentration must be ≥ 50% fT > MIC, and for CTZ, the tissue concentration must be ≥ 60% fT > MIC. In our study, these criteria were met in only 67% of patients treated with AMC and 55% of patients with CTZ. However, it was very difficult from the point of macro- and microcirculation parameters to subanalyse patients achieving or non-achieving appropriate MIC of ATBs in peripheral tissue. The subgroups were very small ((8 patients treated by AMC achieved MIC for more than 50% of evaluation time vs. 4 patients non-achieving these levels/similarly 6 patients treated by CTZ achieved MIC for more than 60% of evaluation time vs. 5 patients without sufficient tissue concentrations), and it was impossible to perform appropriate statistical power analyses. Similar situation is when we would like to compare patients achieving or not appropriate plethysmographic parameters including interarm distance.

In our study, ATB levels in serum and tissue, particularly in the AMC group, positively correlated with serum creatinine levels and negatively with weight, BMI, albumin, and glomerular filtration. Similar correlations in connection to AMC treatment were recently documented by Brazil experts (42). Therefore, it is imperative to highlight the importance, during DFI therapy, of tailoring ATB dosage schemes based on factors such as patient weight, BMI, drug distribution, nutrition, and renal functions.

Regarding the relationship between PAD, microcirculatory impairment, ATB availability, and bactericidal activity, we noted significant positive correlations solely between maximum ATB tissue levels and their AUC with arterial flow, as detected in the treated lower limb by OP. Interestingly, neither ABI, TBI, nor TcPO_2_ correlated significantly with measured pharmacokinetic and pharmacodynamic parameters. These original findings on the interconnection between vascular status, ATB availability, and bactericidal activity have yet to be reported in the literature. A study by Raymakers et al. underscored the importance of tissue perfusion (TcPO_2_) in the penetration of CTZ into the skin, muscles, and bones. However, this study did not follow ATB concentrations in real time, and results were based on immediate ATB levels detected from tissue obtained during amputation procedures (33).

In our study, TcPO2, like ABI and TBI, did not correlate with real-time pharmacology data in either AMC or CTZ. This indicates that the macrocirculation status represented by ABI and TBI and the microcirculation status represented by TcPO_2_, are not as important as the volume of blood that reaches the treated foot through the native arterial network and pre-existing capillaries. Our results clearly demonstrate the important role of plethysmography in the diagnosis of vascular changes (exhibiting sensitivity of 73% to 90% and specificity of 77% to 96% for PAD diagnosis) in patients with IDFUs, reinforcing the need for comprehensive vascular examination in podiatric patients at all times (43).

One of the limitations of this study is the small number of patients enrolled. However, microdialysis studies are technically and logistically demanding to perform and generally involve small numbers of participants. Additionally, patients with critical limb-threatening ischemia were excluded from the study, since these patients typically require urgent revascularization procedures or even major amputations.

In summary, ATB treatment of patients with IDFUs proved satisfactory in only half to two-thirds of study participants. The availability and bactericidal activity of ATBs appear to be impaired by vascular changes, particularly by the volume of arterial flow in the treated lower limbs.

## Data availability statement

The raw data supporting the conclusions of this article will be made available by the authors, without undue reservation.

## Ethics statement

The studies involving humans were approved by The ethics committees of the Institute for Clinical and Experimental Medicine and Thomayer University Hospital. The studies were conducted in accordance with the local legislation and institutional requirements. The participants provided their written informed consent to participate in this study.

## Author contributions

VF: Writing – original draft. RJ: Investigation, Writing – review & editing. SA: Methodology, Writing – review & editing. JH: Investigation, Writing – review & editing. VW: Investigation, Writing – review & editing. PB: Methodology, Writing – review & editing. JM: Supervision, Writing – review & editing. PT: Investigation, Methodology, Supervision, Writing – original draft. JP: Methodology, Supervision, Writing – review & editing. MD: Supervision, Writing – review & editing. DS: Supervision, Writing – review & editing. VL: Investigation, Writing – review & editing. MP: Supervision, Writing – review & editing.
